# Seasonal and diel patterns in Black Sea harbour porpoise acoustic activity in 2020–2022

**DOI:** 10.1002/ece3.70182

**Published:** 2024-10-09

**Authors:** Julia Ivanchikova, Nick Tregenza, Dimitar Popov, Galina Meshkova, Romulus‐Marian Paiu, Costin Timofte, Ayaka Amaha Öztürk, Arda M. Tonay, Ayhan Dede, Uğur Özsandıkçı, Natia Kopaliani, Davit Dekanoidze, Zurab Gurielidze, Karina Vishnyakova, Philip S. Hammond, Pavel Gol'din

**Affiliations:** ^1^ Schmalhausen Institute of Zoology National Academy of Sciences of Ukraine Kyiv Ukraine; ^2^ Sea Mammal Research Unit, Scottish Oceans Institute University of St Andrews St Andrews UK; ^3^ Chelonia Limited Mousehole Cornwall UK; ^4^ Green Balkans NGO Plovdiv Bulgaria; ^5^ Department of Zoology, Faculty of Biology Plovdiv University Plovdiv Bulgaria; ^6^ Mare Nostrum NGO Constanta Romania; ^7^ Faculty of Biology Bucharest University Bucharest Romania; ^8^ Faculty of Aquatic Sciences Istanbul University Istanbul Turkey; ^9^ Turkish Marine Research Foundation (TUDAV) Istanbul Turkey; ^10^ Faculty of Fisheries Sinop University Sinop Turkey; ^11^ Institute of Ecology Ilia State University Tbilisi Georgia; ^12^ BioEcoLinks NGO Odesa Ukraine; ^13^ Ukrainian Scientific Centre of Ecology of the Sea Odesa Ukraine

**Keywords:** Black Sea, diel patterns, fish, F‐POD, GAM, harbour porpoise, migrations, movements, prey, seasonal patterns

## Abstract

The Black Sea is a semi‐enclosed inland sea with an unevenly distributed extensive coastal shelf area and anoxic deep waters. It is inhabited by common and bottlenose dolphins, as well as harbour porpoises, all represented by local subspecies. Between September 2020 and October 2022, 19 F‐PODs deployed by research teams from Bulgaria, Georgia, Romania, Türkiye and Ukraine collected data on acoustic activity of Black Sea harbour porpoises. Strong seasonal and diel patterns were found, which varied in three regions. In the south‐eastern part of the Black Sea, harbour porpoise acoustic activity was higher from January to May, with a peak in April. This pattern agrees with the seasonal anchovy migration from the winter spawning grounds in warmer waters in the south‐eastern region to feeding grounds on the productive shallow north‐west shelf. The diel pattern showed strong nocturnal acoustic activity, which is consistent with anchovy vertical migration. Porpoises on the western side of the Black Sea exhibited a bimodal seasonal pattern in acoustic activity, with a larger peak in April and a smaller one in October. Diel activity was primarily nocturnal. On the north‐west shelf, harbour porpoise acoustic activity was mostly recorded during the warm period from April to October. The diel pattern showed activity mainly during daylight with two peaks: a smaller one approximately at dawn and a larger one at dusk. This pattern is similar to the vertical migrations of sprat. Overall, the results of the study were consistent with the prey being an important driver of seasonal and diel dynamics of harbour porpoise acoustic activity.

## Graphical abstract

Acoustic activity of harbour porpoises showed strong seasonal and diel patterns on the whole Black Sea basin scale. Patterns differ in different regions and show higher activity in the South and East during winter and higher activity in the Northwest during summer. These patterns could be driven by seasonal and vertical migrations of the main prey species, as well as other factors.

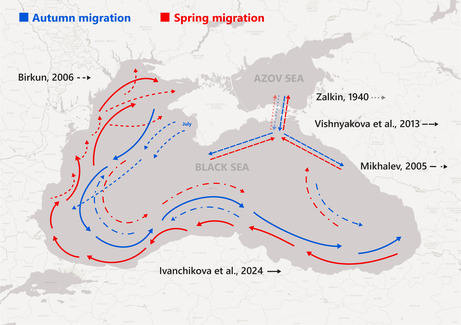

## 1 INTRODUCTION

The harbour porpoise (*Phocoena phocoena*) is atypical among the cetaceans. Distributed in the North Atlantic, North Pacific and Black Sea, it is one of the smallest species and its lifespan is short compared to most other cetaceans (Read, 1995). For example, in the North Atlantic and the Black Sea, harbour porpoises have a maximum age of 20+ years and only a small proportion live beyond 10 years (Gol'din, 2004; Kesselring et al., 2017; Read & Hohn, 1995; Vishnyakova, 2017). Sexual maturity is reached at around 2–6 years of age (Gol'din, 2004; Kesselring et al., 2017; Ólafsdóttir et al., 2003; Webber et al., 2023), which, with a short lifespan, results in a short reproductive lifetime. This is balanced by a short (annual) reproductive cycle, with the interbirth interval close to 1 year (Taylor et al., 2007) – a capability, which is unusual for cetaceans, shared only by porpoises (Hohn et al., 1996), pygmy sperm whales *Kogia* spp. (Bloodworth & Odell, 2008) and minke whales *Balaenoptera acutorostrata* (Perrin et al., 2018; Read, 1990). Harbour porpoises are among the 13 species known to use high‐frequency sonar of relatively narrow bandwidth (Galatius et al., 2019; Moss et al., 2023). Echolocation signals and narrow band auditory filters give them a selective advantage in a coastal environment (Miller & Wahlberg, 2013; Møhl & Andersen, 1973).

The small body size of harbour porpoises and their ‘fast’ life history (Read & Hohn, 1995) requires them to maintain a high rate of energy consumption (Wisniewska et al., 2016) and to dedicate a large proportion of their lives to feeding in order to support their energy budget (Rojano‐Doñate et al., 2018). Harbour porpoises are thus highly dependent on the availability of prey and their foraging behaviour would be expected to be well adapted to finding suitable prey at all times. Such suitable fish prey may undergo seasonal changes in distribution and their availability to porpoises may be further influenced by diel behavioural patterns (Mikkelsen et al., 2013; Ransijn et al., 2021; Schaffeld et al., 2016; Stedt et al., 2023; Todd et al., 2009; Zein et al., 2019).

One would expect, therefore, that harbour porpoise may also show such seasonal and diel variation in distribution and activity. In terms of diel variation in behaviour, studies across their range have found that harbour porpoise activity is mainly nocturnal (Carlstrom, 2005; Clausen et al., 2021; Dracott et al., 2022; Nuuttila et al., 2018; Wingfield et al., 2017). In the Baltic Sea, Sveegaard, Andreasen, et al. (2012), Sveegaard, Nabe‐Nielsen, et al. (2012) have linked seasonal variation in harbour porpoise distribution to their main prey, herring.

This study is focused on the Black Sea harbour porpoise (*Phocoena phocoena relicta* Abel, 1905), which was originally identified as a subspecies based on morphology (Zalkin, 1938) and genetically confirmed (Fontaine et al., 2007; Rosel et al., 1995; Tonay et al., 2017; Viaud‐Martínez et al., 2007). It is one of the smallest cetaceans in the world (Gol'din, 2004), and thus perhaps especially challenged by the need to feed regularly. Similarly to harbour porpoises in other areas, Black Sea harbour porpoises produce narrow‐band high frequency (NBHF) clicks (Au et al., 1999; Dede et al., 2014), which the porpoises use for echolocation and feeding as well as possibly for communication (Sørensen et al., 2018).

Almost a century ago, the diet of the Black Sea harbour porpoise was found to differ seasonally and regionally (Zalkin, 1940). Benthic fish predominated in the summer and winter diet of harbour porpoises in the Azov Sea and in adjacent areas, but European anchovy (*Engraulis encrasicolus*) prevailed in the stomach contents during seasonal migrations of harbour porpoises from the north‐eastern Black Sea through the Kerch Strait to the Azov Sea in spring and back again in autumn (Bilgin et al., 2018; Kleinenberg, 1956; Zalkin, 1940). More recently, European sprat (*Sprattus sprattus*) was found to be the main species in the warm season diet of porpoises in the south‐western and northern Black Sea (Birkun Jr, 2002; Krivokhizhin & Birkun, 2009; Tonay et al., 2007; Tonay & Öz, 1999) Also, whiting (*Merlangius merlangus*) and horse mackerel (*Trachurus* sp.) have been recorded as part of the diet of harbour porpoises in the Black Sea (Bilgin et al., 2018; Bushuev, 2000; Krivokhizhin & Birkun, 2009; Tonay et al., 2007; Tonay & Öz, 1999; Uludüz, 2023; Uludüz et al., 2019).

In this study, we investigate seasonal and diel variation in harbour porpoise acoustic activity at a Black Sea basin wide scale. The aim is both to increase knowledge about harbour porpoise activity in this large area, and to provide an initial understanding of how seasonal and diel activity patterns in Black Sea harbour porpoises may be influenced by the movements and behaviour of key prey species at seasonal and diel scales. While recognising that local acoustic activity may be influenced by different factors, such as human‐induced noise, our aim here is to use an extensive dataset to explore large‐scale patterns at the regional level.

To provide context for our study, we summarise below knowledge about seasonal and diel patterns of movement in the potentially important prey species in the Black Sea. Among these species, European anchovy and European sprat have the greatest, annually fluctuating biomass (Daskalov, 2012; STECF, 2017).

European anchovy prefers temperatures above 6°С and, as autumn temperatures decrease, it migrates from the highly productive shelf waters of the north‐western Black Sea along the coast to the south‐eastern region where water temperatures usually remain above 6°C. In winter, anchovies aggregate in dense groups and undergo vertical migration within the water column to feed on plankton above the thermocline (Chashchin et al., 2015; Daskalov, 2012). They migrate to the surface in the evening, reaching a maximum density before midnight. After sunrise, they aggregate again before returning to the depths. Increasing water temperature, usually in March, signals a migration back to shallow waters of the Azov and northern Black Sea (Guraslan et al., 2017). But overwintering grounds may move temporally as a result of climate variability creating ideal overwintering conditions outside of typical anchovy aggregation locations (Gücü et al., 2017).

European sprat prefers temperatures in the range from 7 to 18°C, and the largest and densest schools are predominantly found in the north‐western part of the Black Sea (Daskalov, 2012; Dubinets & Gubanov, 1988; STECF, 2017). In winter, it mainly concentrates in the open sea where spawning occurs, while during the warm spring and summer seasons it may disperse throughout the coastal shelf zone. During the warm spring and summer season sprat exhibits vertical migrations. After forming dense aggregations in the morning twilight, sprat then spreads in the near‐bottom water layer during the day and concentrates again in the evening twilight, before rising to the upper water layer. Two feeding peaks are observed: a smaller one in the morning twilight and a larger one in the evening twilight. When the water warms and establishes a strong thermocline in spring, sprat tends to stay in the bottom layer in condensed aggregations during the daytime and leaves the bottom to spread higher in the water column but still below the thermocline during the night (Dubinets & Gubanov, 1988).

Whiting (*Merlangius merlangus*) in the Black Sea prefers cooler water layers, at depths of 50–60 m, below the thermocline. It feeds on small fish (sprat, anchovy and juvenile horse mackerel) and therefore may follow sprat in its movements. In the cold season, whiting comes close to shore, and in summer it descends to great depths (Dubinets & Gubanov, 1988). Horse mackerel (*Trachurus mediterraneus*) in the Black Sea, inhabits waters within a wide temperature range, 6–25°C, preferring the warmer end of the range but avoiding riverine estuaries. During summer it stays above the thermocline at around 25–35 m depth to feed. Abrupt changes in thermocline depth during spring and summer, which often happen in coastal waters, can drive horse mackerel movement closer to or further from the coast (Daskalov, 2012; Dubinets & Gubanov, 1988). The other *Trachurus* species *T. trachurus* (Atlantic horse mackerel) is also found in the Black Sea (Daskalov, 2012; Fricke et al., 2007).

The Black Sea harbour porpoise is listed as Endangered on the IUCN Red List (Birkun Jr & Frantzis, 2008). This sub‐species is under pressure from habitat degradation, pollution, noise, overfishing, and, especially, a severe level of bycatch in bottom set gillnets (Popov et al., 2023). In addition, ongoing military operations in the north‐western part of the Black Sea and related underwater blasts might introduce direct threats to animals, as recently found in the Baltic Sea (Siebert et al., 2022).

Seasonal and diel variation in harbour porpoise acoustic activity is interpreted in the context of the movements and behaviour of these key prey at seasonal and diel scales. This new information could be valuable to elaborate conservation policies for cetaceans and management actions to minimise human impact on the species occurring in the Black Sea.

## 2 MATERIALS AND METHODS

### 2.1 Study area: Black Sea

The Black Sea is a semi‐enclosed inland temperate sea with an unevenly distributed extensive coastal shelf area and anoxic deep waters, connected with the Atlantic Ocean through the Marmara, Aegean and Mediterranean Seas via the narrow Istanbul (Bosphorus), Çanakkale (Dardanelles) and Gibraltar straits. The very shallow Azov Sea is connected to the north‐east Black Sea through the Kerch Strait. The Black Sea receives a substantial influx of freshwater, approximately 360 ± 43 km^3^/year (García‐García et al., 2022), from major European rivers such as the Danube, Dnipro, Dniester and also the Don via the Azov Sea. There is no significant tidal activity in the Black Sea.

The Black Sea is characterised by strong stratification of the water column by depth. Due to gradients in temperature and salinity, the water layers are divided by thermocline and halocline, which makes the Black Sea the largest meromictic basin (Sabatino et al., 2020). The anoxic water layer, replete with hydrogen sulphide, renders the main portion of the Black Sea biologically inert, beginning at depths of 100–150 m and extending to its maximum depth of 2210 m (Wegwerth et al., 2018).

**FIGURE 1:**
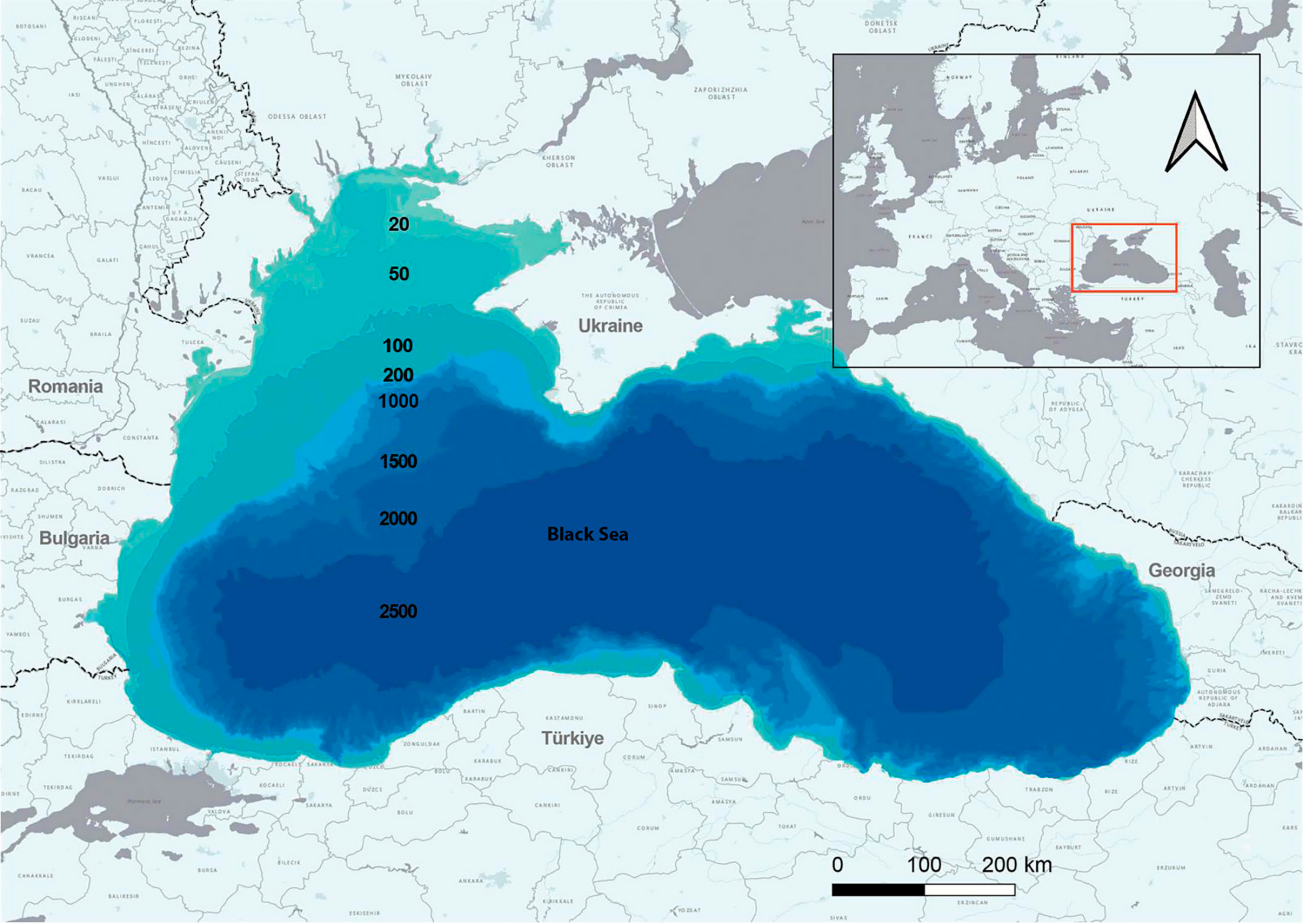
Bathymetry of the Black Sea.

The North‐western shelf area (NWS), with a width of approximately 200 km, forms the greatest part of shelf waters. Outside this and adjoining the western area, the shelf is approximately 20 km wide (Figure 1) (Oguz et al., 2005). NWS is characterised by its shallow depth and the influx of significant freshwater flow – 270 km^3^/year, which accounts for three‐quarters of the total inflow into the Black Sea. The freshwater inflow carries nitrogen compounds and phosphates and contributes to high productivity in the NWS (Zaitsev & Mamaiev, 1997). The annual mean temperature in the NWS varies between 13°C and 14.5°C, and in some winters with exceptionally low temperatures, the coastal waters of the NWS may freeze.

The southern and eastern areas (SE), which stretch from the Istanbul Strait along the southern coast and eastern coast of the Black Sea up to the Kerch Strait, have a narrow shelf that rarely exceeds 15 km in width and a steep shelf edge slope, reaching 2000 m in depth within 30 km from shore. It contains many escarpments and is generally adjacent to mountainous areas (Ross, 1977). These areas of the Black Sea are warmer than the NWS, with annual mean temperatures of 15°C to 16.5°C (Sakalli & Başusta, 2018). Also, the temperature of the Black Sea changes seasonally, with an average July temperature of 24°C in the south and 22–23°C in the north, while during the cold winter season average temperatures near the south and east coast are 6–8°C and in the north they could drop down to 0°C (Shapiro, 2009).

The shelf along the western side of the Black Sea is 25–60 km wide and is characterised by a relatively steep shelf edge slope and, in the north‐western part, the Danube deep‐sea fan, a 150 km long and 10–400 m wide layer of sediments (Popescu et al., 2001). Annual mean temperatures in this part of the Black Sea are 14.5°C to 15°C. The western coastal waters form a ‘transition’ zone between the NWS and the SE areas (Sakalli & Başusta, 2018).

Surface salinity in the NWS is 14‰, while in the SE area it is 18‰, and 16.5‰ in the western side (Oguz et al., 2005). In close proximity to river mouths in the NWS area, the salinity can be as low as 3–10‰ (Zaitsev, 2006).

The water mass of the Black Sea is also stratified by salinity, which increases with depth from 17.5‰ at the surface to 21.9‰ at 100 m depth and then gradually to 22.4‰ at 2000 m (Oguz et al., 2005).

### 2.2 Data collection

The data utilised in this study were obtained under the BlackCeTrends project, an international passive acoustic monitoring initiative aimed at investigating population trends and behaviour of cetaceans in the Black Sea. The project is carried out by research teams from five countries bordering the Black Sea: Bulgaria, Georgia, Romania, Türkiye and Ukraine.

Acoustic data were obtained from F‐POD (Full waveform capture POrpoise Detector) loggers – passive acoustic monitoring instruments (Tregenza et al., 2016), the primary purpose of which is to detect the ultrasound signals (clicks) produced by odontocetes (F‐POD, n.d.). The F‐POD is equipped with an omnidirectional hydrophone that operates in the frequency range of 20 to 160 kHz and captures summarised information about the time‐domain features of selected cetacean clicks. A detailed description of the F‐POD principles is given in the Supporting Information of Ivanchikova and Tregenza (2023).

**FIGURE 2:**
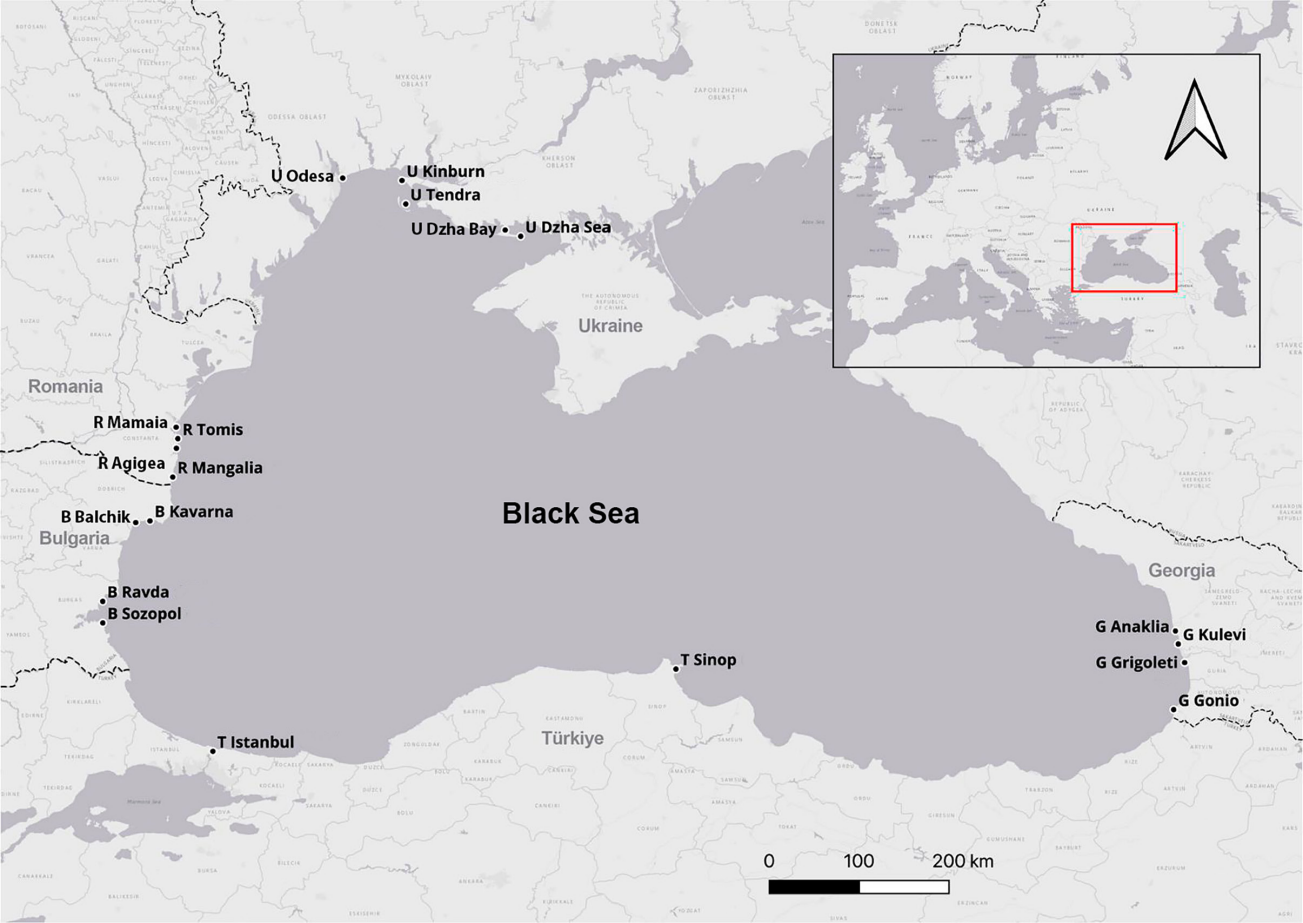
Map of F‐POD locations along the coast of the Black Sea.

Nineteen locations along the coast of the Black Sea were selected for the deployment of loggers, as illustrated in Figure 2. Locations were selected based on logistic and security reasons, as well as knowledge of porpoise presence and preferably low levels of anthropogenic noise generation. All 19 instruments used in this study were brand‐new, received after double calibration according to the standard manufacturing procedure. They were issued with calibration passports, verifying their reliability to function consistently, and were not moved between locations. The F‐POD hydrophones were located mid‐water in the water column. The data were collected from 12 September 2020 to 31 October 2022. Further details regarding F‐POD numbers, locations, depths and seabed types are given in Table S1 Locations in the S1 Supplementary Materials spreadsheet. The exact location of three instruments was corrected during deployment due to loss of equipment and logistical needs, which caused a change in depth in those three locations. The duration of the logged time periods for each location is outlined in Table S2 Gantt (S1 Supplementary Materials). Table S3 FileList (S1 Supplementary Materials) provides a list of all files, along with the number of logged days, and the start and end times of logging.

All the recorded files were processed using the F‐POD custom software; cetacean click trains were extracted with the KERNO‐F v1.0 classifier, which identified all recorded signals in four categories: ‘NBHF’, ‘Other Cetaceans’, ‘Sonar’ and ‘Unclassified’, in four levels of their quality – ‘High’, ‘Moderate’, ‘Low’ and ‘Echo’. We used only ‘High’ and ‘Moderate’ quality click trains identified as ‘NBHF’ to perform our analysis more robustly. To ensure the reliability of the data, each file was cropped to exclude click trains recorded during periods when maintenance boat sonars were active: several hours after the deployment of the POD and several hours before its retrieval for maintenance. Data from the entire 2‐year data collection period were visually validated in order to identify and address any potential errors. Details and results of the validation process are fully described in Ivanchikova and Tregenza (2023).

From the processed F‐POD data, the number of detection positive minutes (DPM) within each hour was obtained for each location. A DPM is defined as a minute in which at least one harbour porpoise click train was logged and is thus a measure of presence of the acoustic activity of animals (Tregenza et al., 2016).

Diel patterns in acoustic activity were investigated using Hour as a covariate (see below). All the F‐PODs logged data in GMT0 time. Because the study area extended 1150 km from west to east (14 degrees of longitude), Hour was converted to astronomical (local) time from GMT. We added 2.0 h for Bulgarian, Romanian, Ukrainian locations and Istanbul, 2.5 h for Sinop in Türkiye (Central Black Sea) and 3.0 h to Georgia locations (Eastern Black Sea).

### 2.3 Data analysis

We used generalised additive models (GAMs) to investigate regional, seasonal and diel patterns of harbour porpoise acoustic activity in the Black Sea. The number of detection positive minutes (DPM) per hour was used as the response variable, with a log link function and error structure described by the Tweedie distribution to account for the strong zero‐inflation and over‐dispersion of the data (Wood, 2006a). How best to stratify the data regionally within the Black Sea was explored by comparing models using several options for dividing the data into different combinations of regions, based on geographical partitioning and connectivity features (see Section 2.3.1 Model implementation).

#### 2.3.1 Model implementation

As a first stage of analysis, we built GAMs to investigate the most appropriate way to stratify the data regionally. First, the whole dataset (including data from all 19 locations) was modelled as one region. F‐POD locations were then grouped into a range of possible regions. The first grouping included two regions – Northwest (Ukraine, Romania) and South (Türkiye, Georgia), with Bulgaria assigned to either the Northwest or the South region. The second level of grouping divided the data into different combinations of three regions:

1. South‐East (Georgia + Sinop + Istanbul), West (Bulgaria + Romania) and Northwest (Ukraine).
2. South‐East (Georgia + Sinop + Istanbul), West (Bulgaria) and Northwest (Romania + Ukraine).
3. South‐East (Georgia + Sinop), West (Istanbul) and Northwest (Bulgaria + Romania + Ukraine).

Finally, dividing the data into four regions was considered:

1. South‐East (Georgia + Sinop), Istanbul (Istanbul Strait), West (Bulgaria) and Northwest (Romania + Ukraine).
2. East (Georgia), South (Sinop + Istanbul), West (Bulgaria) and Northwest (Romania + Ukraine).

In this exploration of regional stratification, separate models of seasonal variation and diel variation were fitted. For seasonal models, DPM was modelled as a function of Month as a cyclic smooth with Region as an additive factor and Hour as a candidate additive factor. For diel models, DPM was modelled as a function of Hour as a cyclic smooth with Region as an additive factor and Month as a candidate additive factor. The full description of regional models is given in S2 Supplementary Materials, spreadsheets ‘Region Selection Month’ and ‘Region Selection Hour’. The regional stratification which received the most support from the data was chosen based on AIC (Akaike, 1974). The second stage of analysis involved fitting seasonal and diel models for each of the chosen regions separately.

For investigating seasonal patterns, DPM was modelled as a function of Month as a cyclic smooth (Wood, 2006b). Hour and Location (serial number of each F‐POD to account for variation among different F‐PODs within a Region) were considered as additive candidate factors. Equivalent models for investigating diel patterns were fitted in which DPM was modelled as a function of Hour as a cyclic smooth, with Month and Location considered as additive candidate factors.

Final model selection was based on AIC. All models were fitted using the mgcv library (Wood, 2006b) within R (R Core Team, 2022), implemented in R Studio (RStudio, 2022).

## 3 RESULTS

**FIGURE 3:**
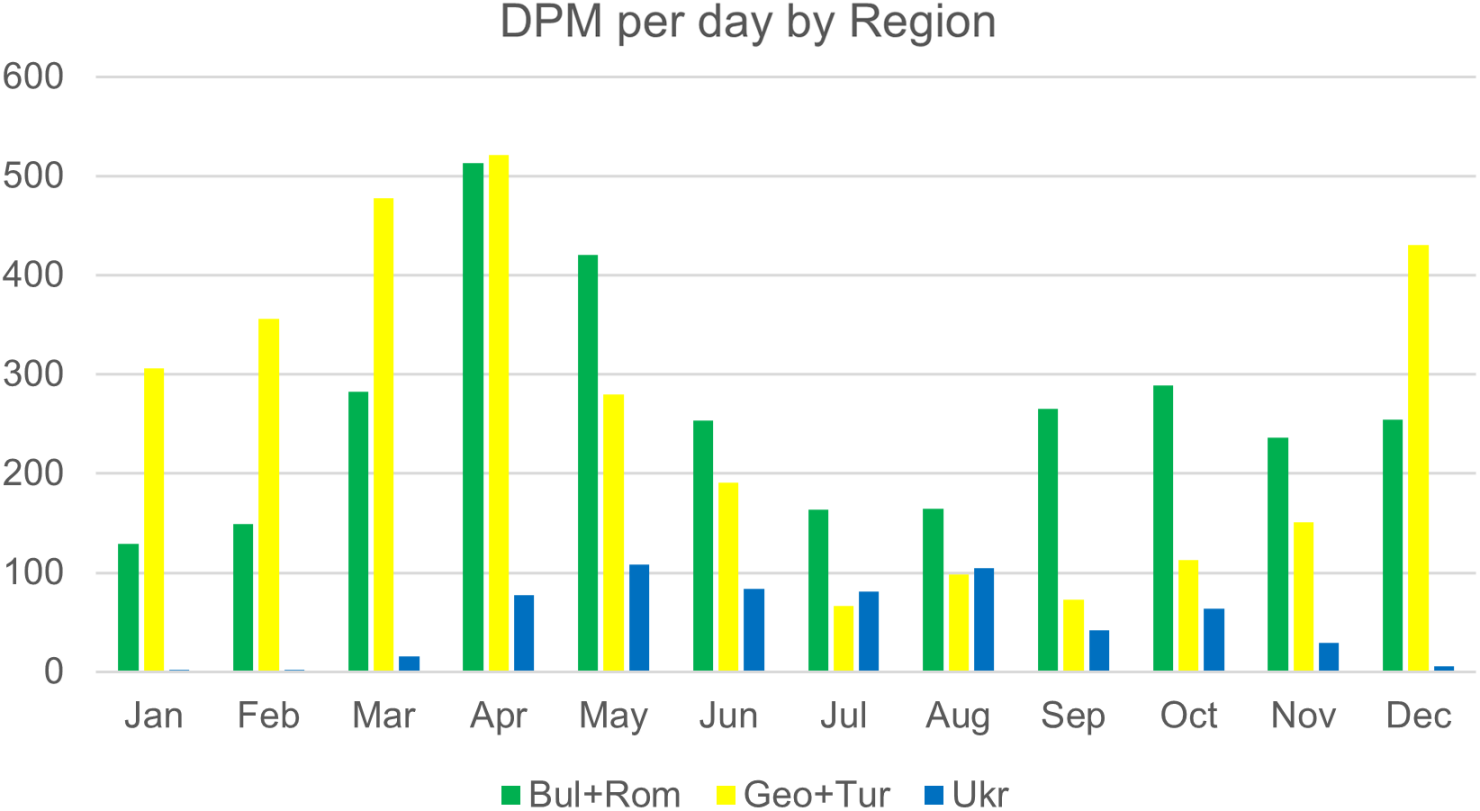
Monthly variation in detection positive minutes per day during 2020–2022 in the Black Sea, grouped into three regions (South‐East – Georgia and Türkiye, West – Bulgaria and Romania, North‐West – Ukraine). The histograms are the sums of mean daily detection positive minutes divided by the number of logged days per month, grouped into the three regions indicated by the results of the regional selection modelling process.

The dataset used for this study as a part of the BlackCeTrends project totalled 87 files, with logging durations ranging from 7 to 163 days. A list of files with details of their duration and seasonal coverage is provided in the S1 Supplement Material: Table S2 Gannt and Table S3 File list. The dataset included a total of 1,312,076 DPMs associated with harbour porpoises in 154,052.4 h of recordings, equal to 6418.85 logdays (or 17.59 logging years). These DPMs were spread unevenly across the 19 locations along the coast of the five riparian countries of the Black Sea (Bulgaria, Georgia, Romania, Türkiye and Ukraine) (Figure 3).

### 3.1 Region selection

The best supported seasonal and diel models of the whole dataset (lowest AIC) were for three regions:

1. South‐East (Georgia + Türkiye).
2. West (Bulgaria + Romania).
3. North‐West (Ukraine).

The next most supported seasonal model had a very large Δ‐AIC of 6176.9 and the next most supported diel model had a very large Δ‐AIC of 3598.9. Details of the region selection process and results are given in the S2 Supplementary Materials, spreadsheets ‘Region Selection Month’ and ‘Region Selection Hour’.

### 3.2 Seasonal patterns

**FIGURE 4:**
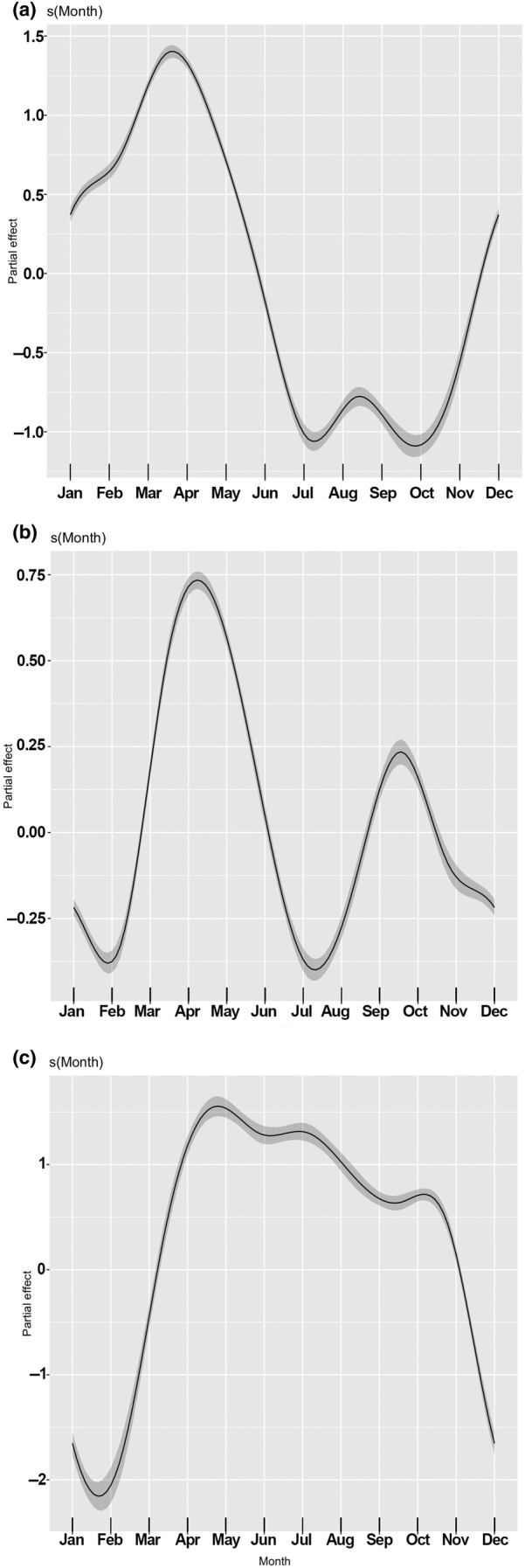
Seasonal pattern of acoustic activity of Black Sea harbour porpoise in the (a) South‐East region (b) West region (c) North‐West region, shown as the partial effect of Month on detection positive minutes (DPM). The grey band around the fitted smooth function represents the 95% confidence interval.

The most supported seasonal model in the South‐East region included Month as a cyclic smooth with Hour and Location as factors. It explained 36% of the deviance in harbour porpoise acoustic activity. The next model had a Δ‐AIC of 618.7 (S2 Supp. Mat. Spreadsheet *South‐East Region*). Acoustic activity of harbour porpoises in this region of the Black Sea mostly occurred from December to May, with the highest activity peaking in April (Figure 4a), following which there was a marked decline in activity during the summer months. In September, there was a slight increase in activity, which then dropped again in October.

The most supported seasonal model for the West region also included Hour and Location as factors; however, this model explained only 12.7% of the deviance in acoustic activity. The next model had a Δ‐AIC of 770.3 (S2 Supp. Mat. Spreadsheet *West Region*). Two distinct peaks of activity were seen: a larger peak in April–May and a smaller one in October (Figure 4b). During the middle of the summer, the activity decreased to a level similar to that in winter.

The most supported seasonal model for the North‐West region also included Hour and Location as factors, with 25.4% of the deviance explained by the model. The next model had a Δ‐AIC of 33.98 (S2 Supp. Mat. Spreadsheet *North‐West Region*). The best model described a strong seasonal pattern (Figure 4c). From the middle of November to the end of February, the acoustic activity of harbour porpoises in this region of the Black Sea was extremely low. In contrast, there was high activity from April to October. The first part of November and the entire month of March showed a marked decrease and increase, respectively, as transitional periods.

### 3.3 Diel patterns

**FIGURE 5:**
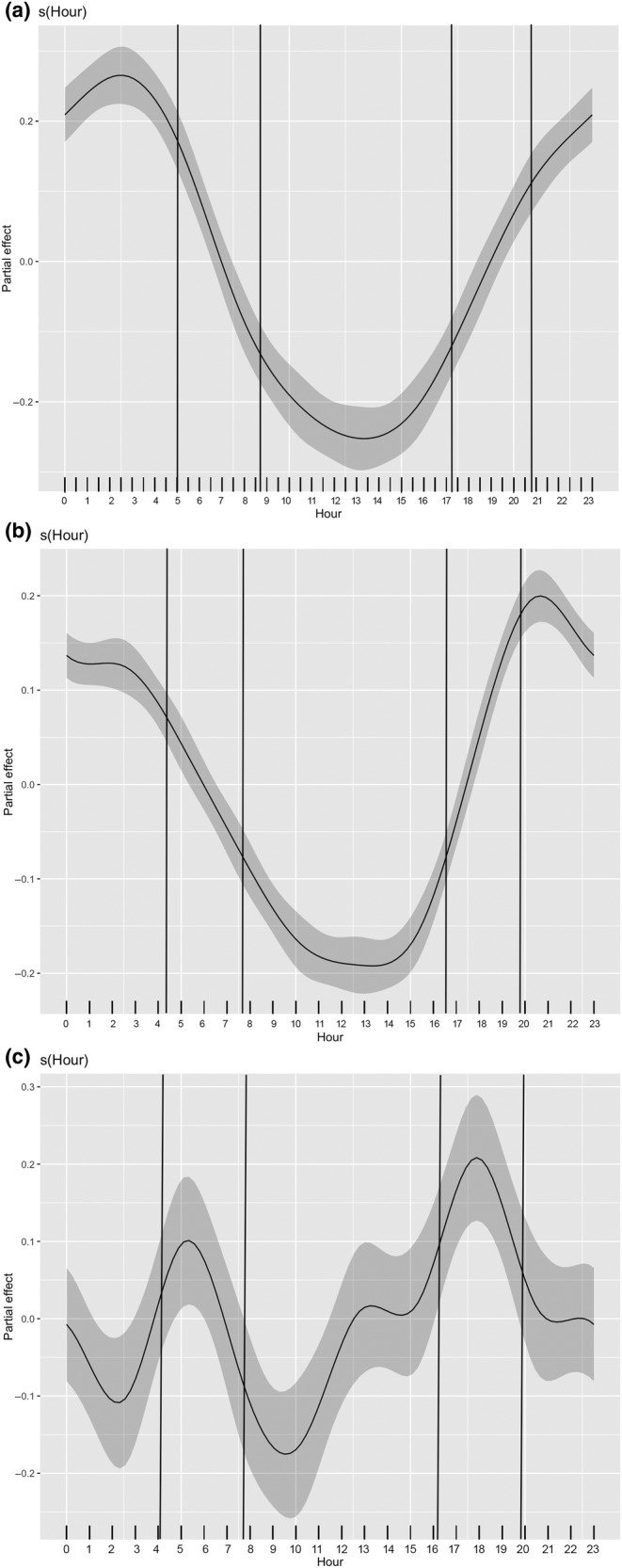
Diel pattern of acoustic activity of Black Sea harbour porpoise in the (a) South‐East region (GMT + 3) (b) West region (GMT + 2) (c) North‐West region (GMT + 2), shown as the partial effect of Hour on DPM. The grey band around the fitted smooth function represents the 95% confidence interval. To provide context in terms of seasonal variation in daylight, the vertical black lines indicate the earliest and latest time of sunrise and sunset in the region, which were: (a) 05:02–08:37 and 17:09–20:51 in the South‐East region; (b) 04:22–07:38 and 16:30–19:52 in the West region and (c) 04:04–07:38 and 16:13–19:53 in the North‐West region.

The most supported diel models included Month and Location as factors. The most supported diel model for the South‐East region explained 36% of the deviance in the data. The next model had Δ‐AIC of 8839.6 (S2 Supp. Mat. Spreadsheet *South‐East Region*). The modelled pattern in the South‐East region (Figure 5a) revealed predominantly nocturnal acoustic activity of harbour porpoises. Activity levels declined after 05:00 GMT + 3, reaching a minimum between 10:00 and 16:00 GMT + 3. Activity levels then increased again during the rest of the day. A plateau of activity was observed in the early morning between 24:00 and 04:00 GMT + 3.

The best supported diel model for the West region explained only 13.5% of the deviance in the data. The next model had Δ‐AIC of 6087.3 (S2 Supp. Mat. Spreadsheet *West Region*). Modelled diel activity of porpoises in the Western Black Sea showed similarities to the patterns seen in the South‐East region, with a higher level of activity during the night and reduced activity during the day (Figure 5b). However, in the West, there was a distinct peak in evening activity at 20:00 GMT + 2. After 22:00, activity declined slightly to a plateau for 2–3 h and then gradually decreased until reaching the minimum range in activity from 10:00 to 15:00 GMT + 2.

The most supported diel model in the North‐West region explained 26% of the deviance in the data. The next model had Δ‐AIC of 1819.96 (S2 Supp. Mat. Spreadsheet *North‐West Region*). Unlike the other two regions, the modelled acoustic activity of Black Sea harbour porpoises in the North‐western region showed two distinct peaks in activity, occurring around 05:00 and 18:00 GMT + 2, with minimum activity at around 10:00 and 24:00 GMT + 2 (Figure 5c).

## 4 DISCUSSION

The analysis of acoustic activity of harbour porpoises in different regions of the Black Sea revealed strong seasonal patterns. In eastern and southern areas, specifically in Georgia and Türkiye, porpoises were mostly active from December until May. In contrast, porpoises in Ukrainian waters of the north‐western part of the Black Sea showed a completely different seasonal pattern. In this region, activity occurred mostly from April to October, covering the warm time of the year, while during the cold winter porpoises were only sporadically recorded. In the western part of the Black Sea, encompassing Bulgaria and Romania, porpoises were recorded throughout the year but displayed a bimodal seasonal pattern of acoustic activity (mentioned as well by Paiu et al., 2022), characterised by a strong peak during April–May and a weaker peak in September. Such a pattern, together with a lower, compared to other models, percentage of deviance explained (12.7%), suggests a transition between the two extreme patterns observed in the seasonally contrasting eastern/southern and north‐western parts of the Black Sea, and/or an influence of other possible factors.

Diel patterns revealed mostly nocturnal activity of harbour porpoises in the south‐eastern and western regions, in contrast to the north‐western area, where acoustic activity was characterised by peaks in activity around dawn and dusk. In the North‐West region, the peak in activity centred around 05:00 which is somewhat after sunrise in June and before sunrise in October (and the peak in activity centred around 18:00 is also somewhat before or after sunset depending on the season).

In this analysis, Hour is effectively a proxy for the amount of daylight, without taking seasonal variation into account. The patterns found are indicative of predominantly nighttime activity in the south‐eastern and western regions but of higher activity around dawn and dusk in the north‐western region. We make no inferences about dependency of harbour porpoise acoustic behaviour on time of day; future work will explore the effect of daylight, and moonlight, as part of a more detailed exploration of harbour porpoise activity in each region. Notably, despite the placement of loggers in relatively shallow depths – less than 25 m, numerous local short‐term shifts in thermocline with abrupt changes in temperature were logged by F‐PODs at several locations. Therefore, given that thermocline movement may hypothetically cause the vertical migration of anchovy and sprat, modelling the influence of temperature on porpoise activity deserves further attention and will also be explored in future work.

### 4.1 Regional, seasonal and diel patterns: Population differences or migration?

Seasonal migrations of harbour porpoise in the Black Sea have been documented several times in the 20th and 21st century (Gol'din, 2004; Salnikov, 1967). Zalkin (1940) suggested movements of porpoises between the Azov Sea and north‐eastern and south‐eastern areas. Vishnyakova et al. (2013) even suggested a more subtle difference between two (sub)populations in that area: one seasonally moving between the Azov Sea and the north‐eastern Black Sea, and the other migrating between the north‐eastern Black Sea and areas to the south of it in winter. These two populations are morphologically distinct from one another (Gol'din & Vishnyakova, 2016) but not genetically (Ben Chehida et al., 2020). Birkun (2006) suggested a seasonal exchange between the north‐western and south‐western areas – a spring movement from the south to northern summer grounds. However, the most recent aerial survey conducted in summer 2019 (Paiu et al., 2024) found higher density of porpoises in the (south‐) western area and lower density in the north‐western and south‐eastern areas.

**FIGURE 6:**
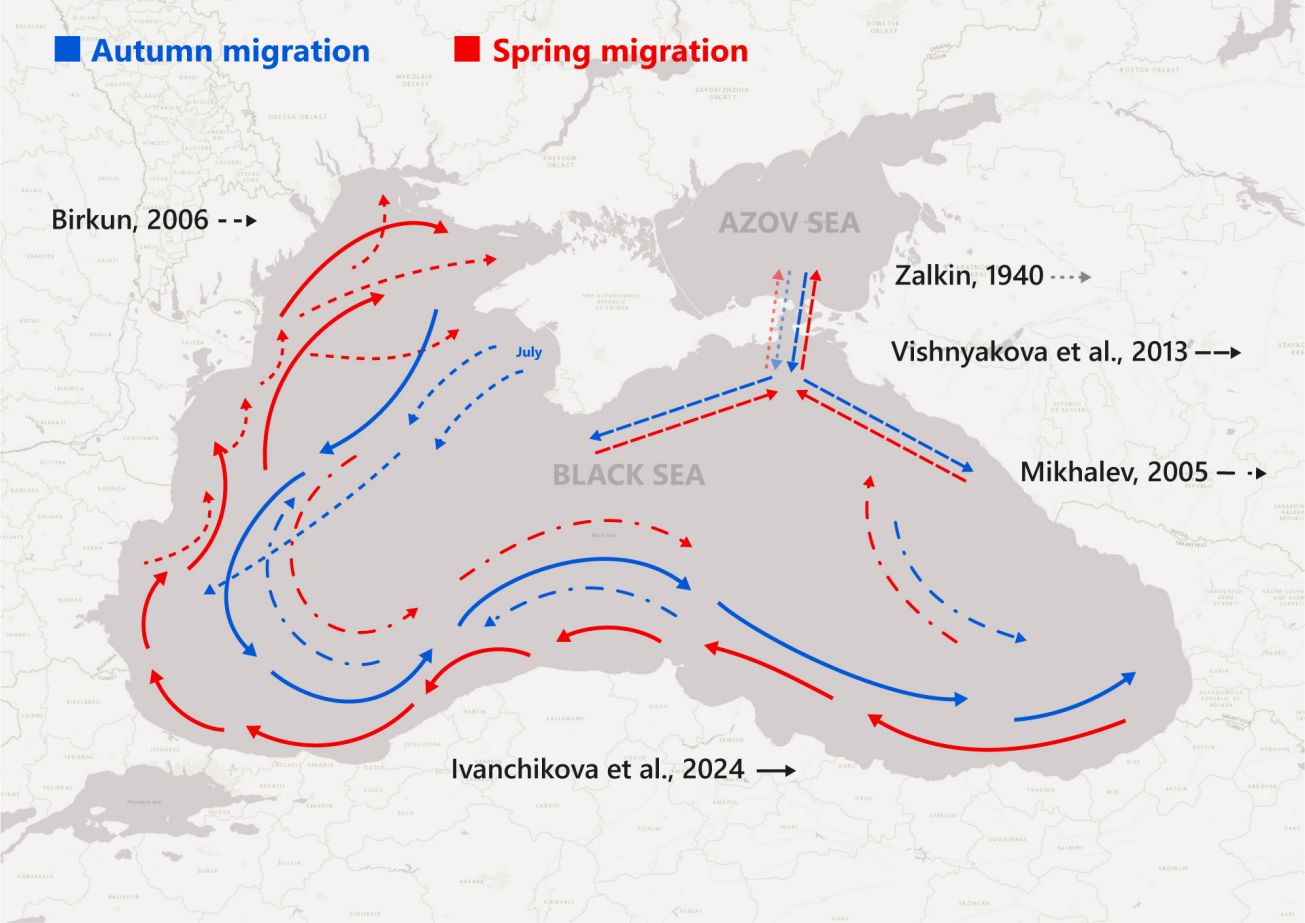
Illustration of different hypotheses of harbour porpoise movements around the Black Sea: Zalkin (1940) described migrations from the Azov Sea to the Black Sea and back, Vishnyakova et al. (2013) proposed different movements of groups of porpoises out of the Sea of Azov, Mikhalev (2005) described movements of porpoises from the north‐east to the south‐east, then to the south west and back, while Birkun (2006) described migrations on the western side of the Black Sea. The possible route of migration of harbour porpoises along Black Sea proper proposed based on our results is also shown.

Salnikov (1967) and Birkun (2006) described a strong seasonal pattern of migrations in the north‐western Black Sea. Cetaceans, mostly porpoises, visited the area to the north of the Danube Delta and the Tarkhankut Cape only between April and October and reached the northernmost Ukrainian coastal waters mostly during the summer. Birkun (2006) believed that some porpoises might move clockwise around the area; from Bulgarian waters to the northernmost areas during the spring followed by south‐eastward movement along the Crimean coast in autumn. Our results summarised above are consistent with the hypothesis of clockwise migration of the harbour porpoises but around the entire Black Sea; however, an opposite counter‐clockwise movement of some groups can be equally possible (Figure 6).

Popov (2023) described significant variation of porpoise density in Bulgarian territorial waters between seasons for the period 2017–2022 with a well‐defined peak in spring. Similarly, Özsandıkçı and Özdemir (2023) observed seasonal variation in harbour porpoise abundance with a peak in spring along the coastal waters of Sinop.

An alternative explanation for the regional variation in seasonal patterns of activity found in our results could be that there are a number of separate groups or populations of harbour porpoises within the Black Sea. This was suggested by Mikhalev (2005), who proposed south‐eastern, south‐western and north‐western local (sub)populations based on the aerial surveys in the 1970–80s. However, there is no evidence for genetic, morphological or life history differentiation of porpoises within the Black Sea proper (Ben Chehida et al., 2020; Notarbartolo di Sciara & Tonay, 2021) but only between the Azov and Black Sea (sub)populations (Ben Chehida et al., 2020; Gol'din, 2004; Gol'din & Vishnyakova, 2016) and between the Marmara Sea and Black Sea (Tonay et al., 2017). Prior information pooled with the new results from this study leads to the conclusion that there is permanent presence of porpoises throughout the year in the south‐western Black Sea and there are also seasonally migrating porpoises between the south‐east and north‐west within the basin (Figure 6).

### 4.2 Regional, seasonal and diel patterns: Ecological drivers

Seasonal migration of anchovy can largely explain the seasonal patterns of acoustic activity of porpoises in the South‐East and North‐western regions. The highest levels of activity in the south‐eastern area were during the anchovy's wintering season coinciding with the lowest activity in the north‐western area (Chashchin et al., 2015; Daskalov, 2012). This was reversed during April, when anchovy migrate to the north‐west shelf to feed and spawn. During the wintering season the thermophilic anchovy usually accumulate in schools with the highest density remaining above the thermocline. Despite this, they still undergo vertical migrations at night which was reflected in the mostly nocturnal acoustic activity of porpoises in the south‐eastern and western areas. There is also evidence for a strong seasonal preference of migrating porpoises feeding on anchovy in the north‐eastern Black Sea (Zalkin, 1940), but there are no data from this area in our study.

The distinctive diel pattern of the north‐western porpoises, with peaks at dawn and dusk, might be indicative of vertical migrations of another major prey species – the sprat (Krivokhizhin & Birkun, 2009; Tonay et al., 2007) which aggregates in denser schools at sunrise, just before moving deeper and shows a similar behaviour at sunset by rising to the upper water layer (Dubinets & Gubanov, 1988).

Migration of prey fish and behaviour thus provide a possible explanation for the strong seasonal and, to some extent, diel variation in porpoise activity in the south‐eastern and north‐western areas of the Black Sea. Our findings imply that the movements of porpoises in these regions are driven by movements of anchovy during the winter season, but are also influenced by other possible factors.

The lower impact of seasonality and diurnal patterns on porpoise activity on the western side of the Black Sea could be, *inter alia*, connected with higher variability in prey movements of another important prey of porpoises – the horse mackerel (Uludüz, 2023). Horse mackerel abundance is high in that region but varies annually and with proximity to the continental slope, with the Danube fan providing an abundant source of nutrients attracting fish. A possible explanation is that porpoises were present in the area year‐round with less seasonal variation than in other areas (Figure 3), so that season is a poorer predictor in the West. Similarly, time of day was a poorer predictor in this area.

That distribution may be driven by anchovy migration in winter and sprat migrations in summer has already been proposed a long time ago for another Black Sea cetacean, the common dolphin *Delphinus delphis ponticus* (Kleinenberg, 1956). According to Bushuev (2000), after the decline of the benthic dwelling fishes (e.g. gobies) due to overexploitation, the harbour porpoise became more dependent on stocks of pelagic fishes and therefore more ecologically similar to the common dolphin (Bushuev, 2000).

Physical or hydrological conditions may also influence spatial and temporal activity of porpoises in some specific habitats, presumably through their effect on prey availability. For example, although all our loggers were deployed close to shore in depths of 8–18 m, the southern locations over narrow shelves near the abrupt sea slopes and upwelling zones showed greater acoustic activity of porpoises than those over a wider shelf.

### 4.3 Context and applications

The regionally diverse patterns of acoustic activity across coastal waters of the Black Sea may have conservation implications for different areas. Most of the logger stations were located within recently identified important marine mammal areas (IMMAs) (IMMAs Black Sea, 2021), and any spatiotemporal management measures should be informed by behavioural characteristics of harbour porpoises, as well as information on presence, habitat use and sensitivity to anthropogenic pressures.

Local variation in cetacean activity at each logger station may also be influenced by anthropogenic factors. For example, marine traffic and fishing vessel activity are especially important in the Istanbul Strait where Dede et al. (2014) showed their impact on seasonal and diel patterns of cetacean activity. Other factors could include construction sites (Mamaya, Romania), ports (Tomis, Romania, or Anaklia, Georgia) or intensive tourism (all locations in Bulgaria). The potential impacts of these factors are worth investigating, and some studies have already been conducted in the region (Dede et al., 2014; Öztürk et al., 2019). In a broader context, the local environmental conditions at a logger station introduce variability to the data. Expanding data collection coverage over a larger area is thus essential to comprehend how such local variations may impact patterns of seasonal and diel variation in acoustic activity.

For example, investigation of the persistence of the seasonal pattern in the West region, a bimodal pattern with reduced activity during July and August, could be important. The current ban on gillnet fishing in Bulgaria and Romania (15th April–15th June) is based only on the turbot spawning period and does not take into account the high activity of harbour porpoises through the end of June, so it overlaps with higher bycatch compared to spring (Popov et al., 2020, 2023; Vishnyakova & Gol'din, 2015). Consideration of seasonal and diel patterns of harbour porpoise activity when determining management action for the fishery could help optimise bycatch mitigation.

## AUTHOR CONTRIBUTIONS

**Julia Ivanchikova:** Conceptualization (equal); data curation (equal); formal analysis (equal); investigation (equal); project administration (equal); writing – original draft (lead). **Nick Tregenza:** Conceptualization (equal); data curation (equal); methodology (equal); project administration (equal); resources (equal); software (equal); supervision (equal); validation (equal). **Dimitar Popov:** Data curation (equal); investigation (equal); methodology (equal); resources (equal). **Galina Meshkova:** Data curation (equal); resources (equal). **Romulus‐Marian Paiu:** Data curation (equal); investigation (equal); methodology (equal); project administration (equal); resources (equal). **Costin Timofte:** Data curation (equal); resources (equal). **Ayaka Amaha Öztürk:** Data curation (equal); resources (equal). **Arda M. Tonay:** Data curation (equal); resources (equal). **Ayhan Dede:** Data curation (equal); resources (equal). **Uğur Özsandıkçı:** Data curation (equal); resources (equal). **Natia Kopaliani:** Data curation (equal); resources (equal). **Zurab Gurielidze:** Data curation (equal); resources (equal). **Davit Dekanoidze:** Data curation (equal); resources (equal). **Karina Vishnyakova:** Data curation (equal); project administration (equal); resources (equal); visualization (equal). **Philip S. Hammond:** Conceptualization (equal); formal analysis (equal); investigation (equal); methodology (equal); resources (equal); supervision (equal); writing – review and editing (equal). **Pavel Gol'din:** Conceptualization (equal); formal analysis (equal); investigation (equal); methodology (equal); resources (equal); supervision (equal); writing – original draft (equal).

## CONFLICT OF INTEREST STATEMENT

The authors declare no competing interest.

## Supporting information


Data S1.

## Data Availability

All data used for performing this research is available from the authors for a reasonable request. Raw data from F‐PODs is in the Figshare Repository via https://doi.org/10.25452/figshare.plus.23982288.
